# Transcriptome Analysis of Canine Histiocytic Sarcoma Tumors and Cell Lines Reveals Multiple Targets for Therapy

**DOI:** 10.3390/cancers17060954

**Published:** 2025-03-12

**Authors:** Alexander I. Engleberg, Ya-Ting Yang, Peter Z. Schall, Marilia Takada, Tuddow Thaiwong-Nebelung, Jacquelyn M. Evans, Elaine A. Ostrander, Vilma Yuzbasiyan-Gurkan

**Affiliations:** 1 Department of Small Animal Clinical Sciences, College of Veterinary Medicine, Michigan State University, East Lansing, MI 48823, USA; engleber@msu.edu (A.I.E.); yangyat1@msu.edu (Y.-T.Y.); schallpe@msu.edu (P.Z.S.); mtakada@ufl.edu (M.T.); 2 Department of Human Genetics, Michigan Medicine, Ann Arbor, MI 48109, USA; 3 Department of Small Animal Clinical Sciences, College of Veterinary Medicine, University of Florida, Gainesville, FL 32610, USA; 4 Veterinary Diagnostic Laboratory, College of Veterinary Medicine, Michigan State University, Lansing, MI 48824, USA; thaiwong@msu.edu; 5 Cancer Genetics and Comparative Genomics Branch, National Human Genome Research Institute, Intramural Program of the National Human Genome Research Institute, National Institutes of Health, Bethesda, MD 20892, USA; jme255@cornell.edu (J.M.E.); eostrand@mail.nih.gov (E.A.O.); 6 Baker Institute of Animal Health, Department of Biomedical Sciences, Cornell University, Ithaca, NY 14853, USA; 7 Department of Microbiology, Genetics and Immunology, Michigan State University, East Lansing, MI 48824, USA

**Keywords:** histiocytic sarcoma, dendritic cells, macrophages, RNA sequencing, transcriptome, myeloid cancers, tumor pathways, immunotherapy, immune checkpoints, small-molecule inhibitors, *FOXM1*, *AURKB*, *PLK1*, *E2F*, *PTPN11*

## Abstract

**Background**: Histiocytic sarcoma (HS) is a highly aggressive malignancy characterized by the excessive proliferation of histiocytes in dogs and humans. A subset of dog breeds, including the Bernese Mountain Dog (BMD), show a remarkably high prevalence of HS. Previous work by us and others has identified somatic driver mutations of HS in the *PTPN11* and *KRAS* genes that activate the MAPK pathway in about 60% of canine HS. However, no somatic driver mutations have been identified in the remaining 40%. **Objectives**: Our goals are to study HS in BMDs to gain insight into the molecular pathogenesis of the disease, and identify rational approaches to therapy. Methods: Here, we report our whole transcriptome analysis of 18 well-characterized BMD HS tumor tissues, as well as three HS cell lines. **Results**: Our analysis reveals the significant upregulation of molecular pathways involving the *FOXM1*, *AURKB*, *PLK1*, and *E2F* genes, in HS as well as hemophagocytic HS, providing new information regarding pathways that may be targeted with inhibitors. In addition, we document the expression of multiple checkpoint genes, suggesting the option of treatment with small-molecule inhibitors together with checkpoint inhibitors. Further, we show that the transcriptomes of three canine HS cell lines mirror those of canine patient tumors, further highlighting their potential use in drug discovery and efficacy studies. Finally, we demonstrate, for the first time, that aurora kinase inhibitors are effective in curtailing the growth of HS cells in vitro and show synergism with MAPK inhibition. **Conclusions**: This study provides the most detailed analysis of the canine HS transcriptome to date, highlighting key pathways in its pathogenesis and suggesting new avenues for both single and combination treatment strategies, which may be pertinent to the treatment of human HS.

## Simple Summary

Insights into molecular changes in cancers have revolutionized the treatment of many cancers. Histiocytic sarcoma (HS) is a malignancy of dendritic and macrophage cell lineages that is prevalent in a subset of dog breeds, including the Bernese Mountain Dog (BMD). The disease is both rare and aggressive in humans and dogs, with limited treatment options. Using HS tumor samples from BMDs, as well as HS cell lines, we investigated the HS transcriptome. We report that multiple pathways, including those involving *FOXM1*, *AURKB*, *PLK1*, and *E2F*, are dysregulated in HS and may serve as targets for novel small molecule therapies. Further, we show that several immune checkpoint genes are expressed, with both PD-L1 and PD-L2 being overexpressed. Our findings indicate that both small molecule and immune checkpoint blockade, alone or in combination, may be effective therapies for this challenging disorder in dogs and humans.

## 1. Introduction

Histiocytic sarcoma (HS) is a highly aggressive malignancy characterized by the excessive proliferation of histiocytes, which are cells of the macrophage and dendritic cell lineage. While HS is an uncommon disease in dogs and humans, a subset dog breeds, including Bernese Mountain Dogs, (BMDs), flat-coated retriever, golden retriever, and Rottweiler, show an increased incidence, with BMDs having the highest at approximately 25% [1,2]. Genetic predisposition in BMDs and flat-coated retrievers have been the subject of many studies [3,4,5,6] and, while some genome-wide association studies (GWASs) have indicated the presence of susceptibility loci, specific genes conferring susceptibility have not been identified. However, insights into the drivers of tumorigenesis have been gained through the targeted sequencing of oncogenes, leading to the identification of driver mutations in *PTPN11* (approximately 56% of those tested), *KRAS*, and *NRAS* (approximately 3%) [7,8], pointing to the activation of the MAPK pathway in HS [9]. Studies in canine HS cell lines also demonstrate mutations in *PTPN11* and *KRAS*, indicating the activation of the MAPK pathway, with one cell line (DH82) showing *PTEN* loss and AKT activation [7]. In addition, we previously demonstrated the efficacy of MAPK pathway inhibition by targeted small molecules such as trametinib and dasatinib in cell lines, and mouse models of canine HS providing support for the use of targeted treatments [10,11]. A phase I trial of trametinib has been concluded and a phase II efficacy trial is ongoing [12].

While our knowledge of human and canine HS has significantly increased in the last few years, significant gaps in the understanding of tumorigenesis exist. Driver mutations have not been identified in about 40% of the canine cases, and it is not clear if targeting the MAPK pathway alone will offer effective treatment, even in cases with mutations in the MAPK genes. Current treatment options for HS in dogs show little efficacy; the most effective regimen uses N-(2-chloroethyl)-N’-cyclohexyl-N-nitrosourea (CCNU), which provides a response rate ranging from 29 to 46% and a median survival time of 85–96 days [13,14].

The targeted RNA sequence analysis of canine HS tumors has identified the dysregulation of numerous genes associated with cell cycle activity and cellular proliferation [6,15]. In one of the few instances of whole transcriptome sequencing, Asada et al. identified the upregulation of the MAPK and PI3K/Akt signaling pathways in both tumor samples and HS cell lines [16]. However, this analysis was performed using a small sample size (five dogs) of differing breeds. Herein, to gain further insight into the molecular changes associated with HS, we carried out the whole transcriptome analysis of eighteen well-characterized HS BMD tumor tissues and three HS cell lines.

Our transcriptome analysis reveals that multiple pathways are activated in BMD HS, including AURKB, FOXM1, and PLK1. Further, we show that the canine HS cell line transcriptomes mirror that of the tumors from canine HS patients, allowing for the testing of drug efficacy. We show, here, that targeting the AURKB pathway may be an effective treatment strategy, laying the foundation for further targeted and combinatorial treatment approaches for HS. As many pathways are shared between canine and human cancers, studies of canine cancer biology inform human cancer studies. In addition, companion animals with cancer enable translational studies allowing for the testing of both small molecule as well as immunomodulatory approaches to treatment.

## 2. Materials and Methods

### 2.1. HS Tissue and Blood Sample Extraction, Processing, and Sequencing Methods

HS tumor samples were from the Michigan State University (MSU) BMD DNA and Tissue Repository. Eighteen HS tumor samples with confirmed histopathological diagnosis were used for RNA extraction, utilizing frozen tumor tissue samples. The tumor tissue was divided into two, with one half used for RNA isolation and adjacent tissue fixed in optimal cutting temperature (OTC) embedding media. The percentage of tumor tissue in each sample was estimated to be >80% by histopathology. RNA was extracted using the mirVANA microRNA isolation kit (Invitrogen, Waltham, MA, USA). The quality of extracted RNA was determined using TapeStation assay. The RNA integrity number (RIN) of the samples ranged from 8.3 to 9.6, indicating excellent RNA quality. For normal controls, RNA was extracted from three BMD blood samples and three tissue samples (spleen, liver, and lung) isolated from German Shepherd Dogs (GSDs). Detailed sample information can be found in Table S1.

The RNA sequencing of HS tumor tissue was carried out at the National Institutes of Health (NIH) by the Intramural Sequencing Center. Libraries were prepared using Illumina TruSeq Stranded Total RNA kit with Ribo-zero Globin depletion and sequenced to a minimum of 100 million pairs of 150 bp reads per sample using an Illumina NovaSeq6000 sequencing machine (Illumina, San Diego, CA, USA) [3]. Raw sequence files (fastq) files were downloaded and processed for analysis at MSU.

### 2.2. HS Cell Line Growth, Maintenance, and RNA Preparation

Transcriptomes of the HS cell lines BD and OD derived from BMD cases, PJ derived from a Rottweiler HS case [10,17], and DH82 (ATCC, Manassas, VA, USA, CRL-3590/RRID:CVCL_2018) derived from a golden retriever were also analyzed. The three cell lines were grown to approximately 80% confluence in RPMI 1640 media supplemented with 10% heat-inactivated fetal bovine serum (10100147, ThermoFisher, Waltham, MA, USA), 1% antibiotic-antimycotic 100X (15240062, ThermoFisher), and 0.1% gentamycin (15710064, ThermoFisher). Cells were incubated in 5% CO_2_ at 37 °C, and used for RNA isolation. Both the BD and OD cell lines were derived from tumor samples from individual BMDs with a histopathologically confirmed HS diagnosis. The DH82 cell line is derived from a golden retriever with the hemophagocytic subtype of histiocytic sarcoma (HHS). Peripheral blood monocytes using blood from a mongrel dog were differentiated into dendritic cells using GM-CSF and IL-4, as described [18], and designated DC3F9 (using 70 ng/mL granulocyte-macrophage colony stimulating factor (GM-CSF) (PeproTech, Cranbury, NJ, USA) and 50 ng/mL interleukin-4 (IL-4) (PeproTech) for seven days to obtain immature Mo-DC, as described previously) [18]. RNA was isolated from each cell source using the mirVana microRNA isolation kit (Invitrogen) and confirmed by Tapestation assay to have RIN > 9.0.

RNA library prep sequencing was carried out at the Genomics Core of the Research and Technology Support Facility of MSU. RNA-seq libraries were prepared using the Illumina Stranded mRNA Library Kit and ligated with IDT for Illumina RNA UD Indexes, following the manufacturer’s recommendations. Completed libraries underwent quality control and were quantified using a combination of Qubit dsDNA HS and Agilent (Agilent Technologies, Santa Clara, CA, USA) 4200 TapeStation HS DNA1000 assays. The libraries were pooled in equimolar quantities and quantified using the Invitrogen Collibri Quantification qPCR kit. Pools were loaded onto one or more lanes of an Illumina NovaSeq 6000 SP flow cell and sequenced to obtain 60 million reads per sample in paired-end read format using a NovaSeq v1.5 300 cycle reagent cartridge. Base calling was performed by Illumina Real Time Analysis (RTA) v3.4.4, and the output of RTA was demultiplexed and converted to FastQ format with Illumina Bcl2fastq v2.20.0.

### 2.3. RNA-Seq Analysis

Raw RNA sequence files from HS and both normal tissues and blood samples were submitted to the ROSALIND^®^ online platform for differential gene expression and pathway analysis (https://rosalind.bio, accessed on 15 November 2024). Data were analyzed using a HyperScale architecture developed by ROSALIND, Inc. (Rosalind, San Diego, CA, USA). Reads were trimmed using cutadapt (v3.7, RRID:SCR_011841). Normalization of raw data, quality control, and functional analysis were also performed using the ROSALIND^®^ platform. Quality scores were assessed using FastQC (v0.11.9, RRID:SCR_014583). Sequencing reads were aligned to the CanFam3.1 canine reference genome using STAR (v2.7.10a, RRID:SCR_004463). Individual sample reads were quantified using HTseq (v2.0.0, RRID:SCR_005514) and normalized via Relative Log Expression (RLE) using the DESeq2 (v1.20, RRID:SCR_015687) R library. Read distribution percentages, heatmaps, and MDS plots were generated using rSeQC (v4.0.0, RRID:SCR_005275) during the QC step. DEseq2 was also used to calculate log2-fold changes (log2FC) and *p*-values. Hypergeometric distribution was used to analyze the enrichment of pathways, gene ontology, domain structure, and other ontologies. The topGO R library was used to determine local similarities and dependencies between GO terms in order to perform Elim pruning [19]. Several database sources were utilized for analysis, including Interpro [20], NCBI [21], mSigDB [22,23], REACTOME [24], and WikiPathways [25]. Genes were considered significantly differentially expressed if they had a log2FC value greater than 1.5 and a false discovery rate with a Benjamini–Yekutieli correction of <0.05. Similarly, for pathway analysis, an adjusted *p* < 0.05 indicated significant alteration of that pathway.

A separate analysis was carried out for the cell lines, which were sequenced as a batch at MSU. RNA sequence files from the three canine HS cell lines and one normal dendritic cell strain were similarly uploaded and processed by the ROSALIND^®^ online platform as described above.

### 2.4. Upstream Regulator Analysis

Differential gene expression data were further analyzed with the use of QIAGEN IPA (v2024.01.20, RRID:SCR_008653, QIAGEN Inc., Hilden, Germany, https://digitalinsights.qiagen.com/IPA, accessed on 15 November 2024). Raw gene expression data was exported from the ROSALIND^®^ platform and imported into IPA for additional and confirmatory pathway analysis and to identify HS-associated upstream regulators. Predicted activation or inhibition of a pathway or regulator was determined by z-score based on gene dataset expression. Pathways or regulators were determined “activated” or “inhibited” using a z-score ≥ |2|. Significance was determined by *p*-value overlap of respective dataset genes [26].

### 2.5. Drug Efficacy Studies

Two aurora kinase inhibitors (AURK-Is), AT9283 and BI847325, were tested alone and in combination with trametinib at multiple doses in three canine HS cell lines (BD, OD, and DH82). The AURK-Is were purchased from Selleck Chemicals LLC (Selleck Chemicals, Houston, TX, USA) and trametinib was purchased from Cayman Chemical (Cayman Chemical, Ann Arbor, MI, USA). All three compounds (AT9283, BI847325, and trametinib) were dissolved in DMSO and stored at −20 °C before use.

### 2.6. Cell Viability Assay

The MTS assay (Promega Corp., Madison, WI, USA) was used to determine the half-maximal inhibitory concentration and IC_50_ values of AT9283 and BI847325 on the BD, OD, and DH82 cell lines. Cells were seeded to a 96-well plate with a concentration of approximately 3000/well. After 24 h, cell culture medium was replaced by complete medium with compounds or vehicle control (0.1% DMSO) for 72 h. The cell viability was analyzed by CellTiter 96 Aqueous Non-Radioactive Cell Proliferation Assay (MTS) and determined by the amount of colored formazan dye produced by live cells. The absorbance of formazan dye was measured at wavelength 490 nm, and IC_50_ values were calculated by PRISM Statistical Software (Graph Pad Software Inc., San Diego, CA, USA, v9.2.0., RRID:SCR_002798). Assays to determine IC_50_ values were run in triplicate.

### 2.7. Combination Index (CI)

Cells were simultaneously incubated with two compounds at a fixed ratio (AT9283: Trametinib = 2:1, 1:1, 1:2, or 1:4; BI847325: Trametinib = 1:1, 1:2, 1:4 or 1:8) for 72 h. The synergistic effects of each pair of drugs were determined via isobologram and combination index (CI) analysis using CompuSyn software (v1.0.1, Combosyn, Paramus, NJ, USA, RRID:SCR_022931). The analysis was adapted from the median-principle methods of Chou and Talalay [27]: results of CI < 1, CI = 1, and CI > 1 indicate synergistic, additive, and antagonistic effects, respectively.

## 3. Results

### 3.1. HS Tumor Gene Expression Results

**Figure 1:**
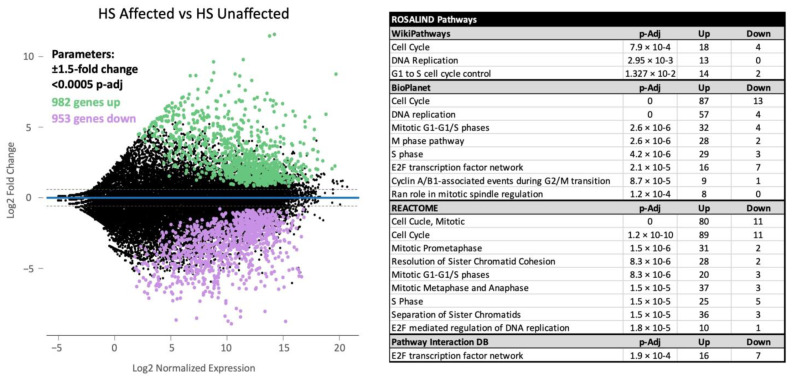
A minus-average (MA) plot and significantly altered pathways for RNA sequence analysis of 18 HS (histiocytic sarcoma)-affected tumors against five normal samples. The analysis yielded 1935 differentially expressed genes (annotated) under parameters of ±1.5 expression log2-fold change (log2FC) and a *p*-adjusted value less than 0.0005, indicated by the dashed lines.Pathway analysis using the ROSALIND^®^ platform identified the significant upregulation of signaling pathways related to the mitotic process.

RNA sequencing was performed on 24 samples comprising 18 HS-affected dogs and 6 unaffected controls. MDS analysis showed the appropriate clustering of most samples based on condition and sample source (Figure S1). One control sample was excluded from further analysis as it was a significant outlier from the other controls. A significance cutoff for gene expression of ±1.5 log2FC and <0.0005 *p*-adjusted value was set to perform differential gene expression analysis. Significance values were chosen to produce an appropriate number (200–3000) of differentially expressed genes for gene set and pathway analysis based on Qiagen Ingenuity Pathway Analysis recommendations. Under these parameters, 2037 genes (1935 annotated genes) were significantly differential expressed, with 982 upregulated and 953 downregulated (Figure 1). Differential gene expression data on all samples are presented in Table S2.

To elucidate the biological effects of this differential expression, significant DEGs underwent pathway analysis, utilizing numerous biological datasets and databases. The top significantly altered molecular and biological pathways are listed in Figure 1. Signaling pathways involving cell cycle entry and maintenance were shown to be significantly dysregulated across various gene sets and databases (REACTOME, BioPlanet, WikiPathways). Gene set analysis utilizing the Pathway Interaction Database identified the significant alteration of specific pathways related to PLK1 signaling events, the E2F transcription factor network, aurora B signaling, and the FOXM1 transcription factor network.

**Figure 2:**
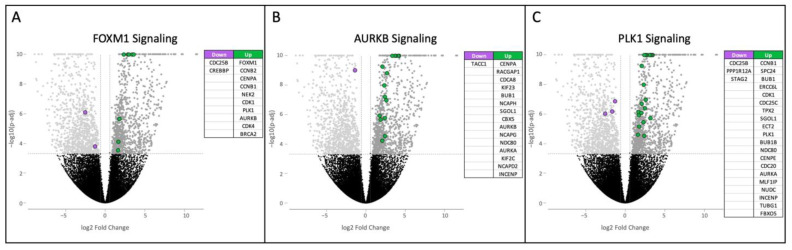
Volcano plots of significantly differentially expressed genes in the (**A**) AURKB, (**B**) FOXM1, and (**C**) PLK Pathway Interaction Database gene sets generated from RNA-Seqanalysis of HS tumors. Upregulation of the three pathways is seen in HS tumor samples compared to normal controls. Gene expression is reported as log2FC and significance is reported as log10 *p*-adjusted value. Significance is achieved with ±1.5 log2FC and a *p*-adjusted value less than 0.0005, shown by the dashed line Upregulated and downregulated genes are colored green and purple, respectively.

Within the FOXM1 pathway, dysregulation is characterized by the overexpression of multiple important cyclins and cyclin-dependent kinases (Figure 2A). Notably, the *FOXM1* gene was highly upregulated (3.39 log2FC). The aurora B signaling pathway exhibited 16 differentially expressed genes, with 15 upregulated and 1 downregulated (Figure 2B). The aurora kinase coding genes *AURKA* and *AURKB* showed overexpression, with AURKB chromosomal passenger complex (CPC)-associated genes (*CDCA8* and *INCENP*) also being significantly upregulated. Significant genes in the PLK1 signaling pathway are presented in Figure 2C, indicating the additional upregulation of the pathway.

To better characterize the pathway and regulator effects of the differential gene expression data, the IPA software (v2024.01.20) package was utilized (Qiagen Inc., Hilden, Germany). The analysis of gene expression through IPA identified numerous dysregulated signaling pathways (Table S3). Signaling pathways related to multiple stages of the cell cycle and mitosis, notably in metaphase and anaphase, are predicted to be activated in the HS-affected samples. Additionally, pathways involved in breast cancer regulation and S100 signaling are predicted to be inhibited or downregulated.

**Figure 3:**
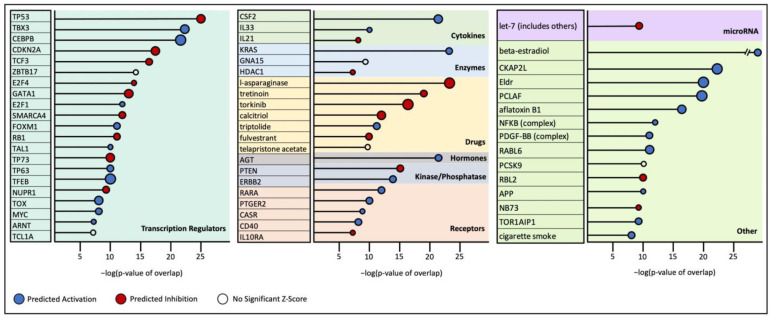
Ingenuity Pathway Analysis predicted activity of upstream regulators in HS tumor samples. Predicted activation (blue) or inhibition (red) of a regulator is determined by a calculated Z-score. Z-score magnitude is depicted in the figure as circles of increasing size. Significance is presented in the log10 transformation of the *p*-value of overlap, comparing dataset genes to genes that are regulated by a particular transcription regulator. Predicted inhibition of the tumor suppressor genes TP53, CDKN2A, RB1, and PTEN is seen in our HS tumor samples.

Upstream regulator analysis revealed numerous significantly altered molecular interactions (Table S4). The top regulators and their predicted state are detailed in Figure 3. Notably, anti-proliferative and apoptotic transcription factors such as, TP53, TP73, CDKN2A, and RB1 were predicted to be significantly inhibited. Conversely, proliferative, anti-apoptotic, and pro-cell-cycle factors such as FOXM1, MYC, TBX3, CEBPB, and E2F1 were predicted to be activated. The predicted activation of the oncogene KRAS, which is an upstream activator of the MAPK signaling pathway, and the predicted inhibition of PTEN, which activates PI3K/Akt signaling, were also observed.

The pathway analysis of the differential expression data also identified the inhibition of numerous drug/chemical pathways. Unsurprisingly, the chemical pathways of FOXM1 and mTOR inhibitors, NB73 and torkinib, respectively, were predicted to be inhibited. In addition, genes targeted by the drug l-asparaginase were predicted to be inhibited.

### 3.2. ROSALIND HS Cell Line Gene Expression Results

RNA sequencing was performed on three canine HS cell lines and one canine normal cell strain, and differential expression analysis was conducted to compare the three affected cell lines with the normal one. A significance cutoff for a gene expression of ±1.5 log2FC and <0.05 *p*-adjusted value was set, with the aim of identifying 200–3000 genes for gene set and pathway analysis. Under these parameters, 1045 genes (987 annotated genes) were significantly differentially expressed, with 565 being upregulated and 422 downregulated (Figure S2). Full gene expression data for this comparison are detailed in Table S5.

The top significantly altered molecular and biological pathways are listed in Table S6. Similar to the HS tumor sequencing results, pathways involving DNA synthesis, replication, and cell cycle phases were shown to be significantly dysregulated across gene set databases. The significant dysregulation of pathways involving PLK1 signaling, the E2F transcription factor network, aurora B signaling, and the FOXM1 transcription factor network was similarly seen in these cell line data. Additionally, aurora A signaling was found to be significantly dysregulated, with all significant dysregulated genes being overexpressed. Gene ontology enrichment analysis also revealed the significant upregulation of genes within the “chromatin remodeling at centromere” gene set. Of note was the fact that both the gene *CENPA* and its co-localization factor *HJURP* were highly overexpressed (4.72 and 5.29 log2FC, respectively) (Table S6).

### 3.3. HS vs. HHS Differential Gene Expression

Among the 18 HS case samples, 3 are from dogs diagnosed with the hemophagocytic histiocytic sarcoma (HHS) subtype of HS. In analyzing samples from each subtype compared to unaffected controls, we found that, while individual gene expression varied, the molecular pathways identified as significantly dysregulated were the same between HS and HHS. In both analyses, molecular pathways related to cell cycle signaling, DNA replication, and stage of mitosis, including the spindle formation, resolution, and separation of sister chromatids, were all significantly upregulated (Tables S7 and S8). Additionally, the AURKB and PLK1 signaling pathways were upregulated. FOXM1 signaling was upregulated, but significance was not reached.

A direct comparison of HS and HHS gene expression profiles yielded numerous differentially expressed genes; however, pathway analysis revealed only one significantly altered cellular signaling pathway relating to cholesterol biosynthesis, which was significantly downregulated in HHS samples compared to HS-only samples and to HS controls.

### 3.4. Transcriptome Comparison of HS Cases with PTPN11 Variant vs. Without PTPN11 Variant

As our previous work has shown that activating mutations in PTPN11 drives MAPK/Ras pathway signaling in HS [7,8], we compared the transcriptome of BMD samples with and without PTPN11 mutations. Of the 18 HS tumor samples, 14 contained PTPN11 activating mutations, and four dogs had no PTPN11 variant. In comparing the gene expression of each group to control dogs, cell cycle activity was predicted to be significantly upregulated, and similar molecular signaling pathways, such as FOXM1, AURKB, PLK1, and E2F, were found to be significantly upregulated (Table S9). In a direct comparison of expression between samples with and without PTPN11 mutations, we identified 275 differentially expressed genes (Table S10). Of note was the fact that the telomerase reverse transcriptase (TERT) gene was differentially expressed (6.41 log2FC), with upregulation in the wild-type PTPN11 tumor samples.

### 3.5. Differential Expression of Immune Checkpoint Genes

Recent immune checkpoint inhibition studies suggest a promising avenue of treatment for numerous cancers and tumor types [28]. Checkpoint inhibitors may also be promising drugs for treating HS, as suggested by the differential expression of numerous immune checkpoints, including CD274 (PDL1) and PDCD1LG2 (PDL2) (Table 1), in this study. Also, while not differentially expressed, the important immune checkpoints PDCD1 (PD1) and CTLA4 were present in all tumor samples.

### 3.6. Effect of AURK Inhibitors

Because of the observation that the AURKB pathway was upregulated in HS samples, we tested the effect of aurora kinase inhibitors (AURK-Is) on HS cell lines. Two AURK-Is, AT9283 and BI847325, were tested in three canine HS cell lines using an MTS assay. As shown in Table 2, the resulting IC_50_ values ranged from 117.5 to 285.7 nM when treated with AT9283. The application of BI847325 resulted in IC_50_ values ranging from 19.7 to 31.4 nM. The Cmax values for each compound are also presented in Table 2, as determined by previous studies [29,30].

**Figure 4:**
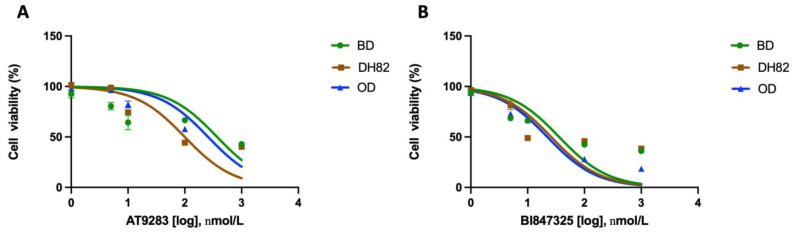
(**A**,**B**) depict the cell viability curves for the HS cell lines BD, OD, and DH82 when treated with the aurora kinase inhibitors AT9283, BI847325, respectively. IC_50_ values for both inhibitors fall below their Cmax values, shown in Table 2, suggesting inhibitory efficacy.

Previously, we showed that the MEK inhibitor trametinib is effective in inhibiting the growth of HS tumor cells both in vivo and in vitro [10,11]. We tested the therapeutic potential of a combined treatment of AT9283 and BI847325 with trametinib. Figure 4 shows the results of combination assays testing different ratio combinations trametinib with AT9283 (Figure 4A) and BI847325 (Figure 4B). Both drug combinations exhibit good pharmacokinetic synergy, with the combination index (CI) being less than one across all cell lines at most of the ratios tested (Figure S3). Full combination assay data are presented in Table S11.

## 4. Discussion

This study provides an extensive exploration of transcriptomic changes in canine HS, especially for HS in BMDs. The transcriptome analysis of 18 HS tissues from BMDs underlines the importance of cell cycle activation in HS, notably in the significant upregulation of molecular pathways involving the *FOXM1*, *AURKB*, *PLK1*, and *E2F* genes. The upregulation of the cell cycle is a hallmark of almost all cancers; however, our data point to these four majors signaling pathways as specifically relating to canine HS. These pathways are similarly dysregulated in the three hemophagocytic HS cases tested, and the analysis of samples with and without a *PTPN11* mutation likewise identified the upregulation of the same four molecular signaling pathways. Additionally, our transcriptomic analysis of the HS cell lines is consistent with that of HS tissues, validating their appropriateness for further studies, especially for drug discovery. We also show, for the first time, that aurora kinase inhibitors are effective in inhibiting the growth of HS cells in vitro, identifying *AURKB* as a potential molecular target in the treatment of HS.

While HS is very rare in humans, the study of Egan et al. profiled the transcriptome of 21 archived primary HS human cases and identified gene set associations relating to the increased activity of cell cycle processes and cellular proliferative pathways [31]. The transcriptional analysis of canine HS, to date, has been limited to targeted sequencing and/or comparisons between only a few samples from multiple breeds [3,6,9,15,16]. However, due to its higher prevalence in dogs than humans, there is an opportunity for larger-scale cohort studies and clinical trials in canines. Utilizing targeted RNA sequencing, a study by Kennedy et al. showed evidence of disruption in DNA replication, repair, and cell cycle/checkpoint regulation processes in canine HS [15]. In a study by Asada et al., the transcriptomic analysis of five canine HS cases across multiple breeds confirmed the importance of ERK and AKT dysregulation in canine HS [16]. Further, in both human [9,31,32,33] and canine [3,7,9,10] HS, special emphasis has been given to the MAPK pathway, with multiple lines of evidence showing pathway upregulation. In humans, some cases of HS develop through trans-differentiation from other types of leukemia and lymphoma, often carrying BRAF^V600E^ mutations, which are rarely seen in canine HS cases [34,35].

Our previous work has shown the phosphorylation of ERK in HS tumor cells, implying the activation of the MAPK pathway in disease [10]. Additionally, in the HS cell line DH82, which is characterized by a PTEN loss, AKT phosphorylation is observed, indicating the activation of the PI3K/Akt pathway [10]. The identification of driver mutations in PTPN11 and KRAS further implicates the dysregulation of both MAPK and PI3K/Akt signaling [7,16]. We showed previously that the inhibition of MAPK signaling by the MEK inhibitor trametinib is a promising avenue of therapy in cell lines and mouse models of canine HS [10,11]. A phase I trial has been successfully completed [12] and a phase II trial is in progress. Yet, it is likely that multiple avenues of treatment will be needed, as our current understanding of HS indicates the involvement of numerous signaling pathways. Additionally, cases of histiocytic neoplasms often are refractory and develop resistance to small-molecule inhibitors [36,37]. The route and efficacy of treatment may also be further influenced by the presence of patient-specific somatic mutations [38].

The targeted RNA sequencing of canine histiocytic malignancies across dog breeds has demonstrated the upregulation of genes involved in double-stranded DNA damage response and mitotic spindle complex assembly (including *AURKA* and *AURKB*), implicating the dysregulation of mitosis in the progression of HS [15]. The current HS transcriptome data support these findings and highlight numerous tumor-associated genes and signaling pathways, adding clarity to the transcriptional profile of HS. In addition, the transcriptome of the three canine HS cell lines analyzed here supports the dysregulation of the same affected signaling pathways (Table S6).

Our data identified the overexpression of key genes related to the mitotic process, thus supporting the dysregulation of the cell cycle as a key driver of HS progression. These include the overexpression of *PLK1*, *AURKB*, *BIRC5* (survivin, 1.23 log2FC), and certain *CENP* isoforms, suggesting that HS tumor cells undergo unrestrained mitosis. A key effector of many of these genes and of the cell cycle is FOXM1. Importantly, FOXM1 is involved in regulating several genes critical to the segregation of chromosomes and cytokinesis at the end of mitosis [39,40,41]. In this study, we find that the overexpression of the FOXM1 gene by a 3.5 log2FC in the HS tumor samples occurs in parallel with an increase in the expression of numerous cell-cycle- and mitosis-associated transcription targets of FOXM1. FOXM1, having a wide range of interactions, also participates in a positive feedback loop utilizing the RAS/MAPK pathway [42]. As FOXM1 is also a key component of cell division and replication, its overexpression is observed in many cancer types [43,44,45,46]. However, because FOXM1 is a transcription factor with wide-ranging targets and is primarily located in the nucleosome, it has not been a successful drug target. However, our upstream regulator analysis identified the drug interaction pathway of the FOXM1 inhibitor NB73 as significantly inhibited, thus suggesting a new route for treatment. Indeed, NB73 has been shown to be effective in inhibiting FOXM1 in myeloma cells in both cell culture and a xenograft mouse model [47].

As seen in Table S3, one of the other top pathways associated with HS is AURKB. The aurora kinase family of genes has three paralogs in humans that are conserved in dogs: aurora kinases A, B, and C (AURKA, AURKB, AURKC) [48,49,50]. The aurora kinases function as protein kinases involved in the progression of the cell cycle [50]. AURKB, specifically, is a regulatory target of FOXM1 and is a main driver of both mitotic metaphase and anaphase [51,52,53], specifically forming a chromosome–passenger complex (CPC) with INCENP, survivin, and borealin, and performs a key role in regulating several processes such as chromosome segregation, cytokinesis, protein localization to the centromere, microtubule–kinetochore attachment, and the regulation of the mitotic checkpoint [54,55,56,57]. In our expression data, AURKB signaling is significantly upregulated in HS samples. Transcriptionally, BIRC5, CDCA8, INCENP, AURKA, and AURKB are also upregulated (Table S2). The overexpression of AURKB has been shown to cause aneuploidy and chromosome instability in vitro in various cancer types [58,59].

In addition to increased activity of mitotic processes, the dysregulation of cell cycle checkpoints was observed, with the DEG analysis revealing the increased gene expression of major cell cycle regulation targets of FOXM1, such as CCNB2, CDC25A, and PLK1 (Table S2). PLK1 also interacts with FOXM1 in a PLK-dependent phosphorylation of FOXM1 that creates a positive feedback loop, increasing the transcriptional activity of FOXM1 and of PLK1 itself [41]. The serine/threonine protein kinase, CDK1, was also found to be upregulated (2.8 log2FC). CDK1 is essential to the FOXM1-PLK interaction by phosphorylating FOXM1, thus facilitating interaction with PLK1 [41]. PLK1 also has a key mitotic interaction with the protein survivin (BIRC5), which, as described previously, forms part of the mitotic CPC and is essential to AURKB signaling and mitotic progression [57,60]. Multiple small molecules targeting CDK1 are under development [61].

The data presented here show that, in addition to FOXM1, targeting AURKB and PLK1 may curb the increased cell proliferative activity of cancer cells. Both proteins have been previously targeted in human cancers, with multiple AURKB and PLK1 inhibitors gaining FDA approval [62,63,64,65,66]. However, AURKB and PLK1 inhibition has not yet been tested in the treatment of human or canine HS. In this study, we tested the efficacy of two aurora kinase inhibitors (AURK-Is) in three canine HS cell lines in combination with the MEK inhibitor, trametinib. As noted in Table 2, the IC_50_ values of both tested AURK-Is fall below the maximum achievable plasma concentration (Cmax) reported values of 3356 nM and 500 nM for AT9283 [29] and BI847325 [30], respectively. This finding strongly supports the feasibility of further studies to study the efficacy of these drugs in the clinic. Further, we demonstrate that the tested AURK-Is show synergistic effects with trametinib in cell lines (Figure S3). Notably, the drug AT9283 was brought to clinical trial for the treatment of acute leukemia in humans, but was terminated early due to limitations with poor recruitment and significant clinical adverse effects [67]. Our upstream regulator analysis also identified the AURK-I Tozasertib as a potentially effective inhibitor of HS cell growth and proliferation.

In a recent study, we showed that variant profiles in the PTPN11 and KRAS genes of HHS-affected dogs is similar to that observed in HS samples, suggesting the dysregulation of common signaling pathways in both cancer types [8]. Gene expression data from this study support this view. While HHS has a slightly different cell lineage than HS, these data demonstrate that HS and HHS can be approached with similar treatments.

One significantly differentially expressed gene between the canine HS tumors with and without PTPN11 mutations was TERT. The upregulation of TERT may be a key driver of tumorigenesis in HS without PTPN11 mutations. TERT overexpression has been experimentally shown to be essential to the immortalization and malignant transformation of cells, and multiple mechanisms, primarily epigenetic changes, are proposed to alter its expression [68]. Several strategies are in development to inhibit TERT function, ranging from small molecules to vaccines, as reviewed by Waksal et al. [69].

Numerous inhibitory immune checkpoint genes are differentially expressed, as shown in Table 1; but, importantly, all of the genes are constitutively expressed in all tumor samples, strongly suggesting that immune checkpoint inhibitors may be effective in HS treatment, including anti-PD1, anti-CTLA4, and anti-Lag3. Specifically, PD1 is significantly overexpressed. PDCD1 expression in canine HS has also been reported by Lenz et al. [70]. Additionally, a canine anti-PD1 antibody (Gilvetmab) is now commercially available [71]. In addition, those targeting CTLA4 [72] and LAG3 [73] are under development. Future studies with immune checkpoint inhibition alone and in combination with small molecules like trametinib are warranted.

Genome-wide association studies have identified a locus near the tumor suppressor CDKN2A to be associated with susceptibility to HS in BMD [5]. However, the same study has shown the relative increased expression of CDKN2A in histiocytes from HS cases, indicating that it is not necessarily through loss or the decreased expression of CDKN2A that this pathway is altered or suppressed in susceptible dogs. In the current study, CDKN2A expression is similar between HS and normal tissues. But, the pathway analysis points to the significant downregulation of the CDKN2A pathway. In human HS, the loss of CDKN2A is among the most frequently observed changes in tumors, with one study reporting a loss in 50% of the 28 cases accumulated over an 18-year period [33].

Other upstream regulators predicted to be significantly activated include KRAS and MYC, both important proliferative signals. While only a few cases of HS have been shown to carry KRAS mutations, the activity of this pathway is clearly important across HS tumors [31,32,33]. Our data also reveal the predicted inhibition of the regulators *CDKN2A/B*, *RB1*, and *PTEN*, which have been shown to be significantly dysregulated, often with copy loss or deletion, in numerous canine HS cases [4,15].

## 5. Conclusions

Histiocytic sarcoma is a highly aggressive cancer with limited therapeutic options. The current study reveals a far more complete portrait of HS cancers than reported previously. Both pathways and genes of relevance are revealed, suggesting not only avenues for further study as it pertains to clinical care, but also providing a foundation for further cancer cell biology studies. The fact that many of the same genes and pathways are implicated in human and canine cancers highlights the importance of studying spontaneous cancers in dogs for purposes of basic discovery and evaluation of new therapies. Numerous dysregulated signaling pathways that are candidates for therapeutic intervention have now been identified, including for HS case that lack driver mutations in the MAPK pathway. We demonstrate the synergism of aurora kinase inhibition with the MAPK pathway inhibitor trametinib in curtailing HS cell growth. Additionally, cancers in pet dogs allow for the evaluation of immunomodulatory approaches to cancer treatment, as they have an intact immune system. Although further studies are needed, we are now in an era where we can pursue multiple options for the development of rational treatments for canine HS, which has, so far, been intractable, and which, in turn, can inform translational studies for humans.

## Figures and Tables

**Table 1:** Expression of inhibitory immune checkpoint genes in canine HS tumors.

Inhibitory Molecule					
Alias	Gene Name	Average Log2 Expression	Average Log2 Fold Change	Significance (*p*-adj)	Compared to Control
A2AR	ADORA2A	10.87	−1.84	1.78 × 10^−3^	Down
A2BR	ADORA2B	9.25	0.711	N.S.	
PD-L1	CD274	13.64	2.36	1.84 × 10^−4^	Up
PD-L2	PDCD1LG2	12.61	2.39	2.62 × 10^−4^	Up
PD-1	PDCD1	8.50	0.128	N.S.	
TIM3	HAVCR2	8.73	0.145	N.S.	
VISTA	VSIR	14.27	−3.14	7.31 × 10^−7^	Down
LAG3	LAG3	10.71	0.497	N.S.	
IDO	IDO1	14.08	0.646	N.S.	
CTLA-4	CTLA4	10.55	0.264	N.S.	
BTLA	BTLA	11.10	−3.46	1.7 × 10^−4^	Down
B7-H4	VTCN1	7.17	−3.33	1.08 × 10^−3^	Down
B7-H3	CD276	12.98	2.45	9.01 × 10^−5^	Up

**Table 2:** Pharmacokinetic data and IC_50s_ for the aurora kinase inhibitors AT9283 and BI847325.

	AT9283	BI847325
BD (IC50)	285.7 nM	31.4 nM
DH82 (IC50)	117.5 nM	19.7 nM
OD (IC50)	269.1 nM	23.8 nM
C_max_	488.2 nM	500 nM (in mice)
Molecular Targets	Aurora A/B JAK2/3 ABL FLT3	Aurora C MEK 2

## Data Availability

The raw RNA sequence data presented in this study have been deposited in NCBI’s Gene Expression Omnibus (GEO) and are accessible through GEO Series accession number GSE288068: https://www.ncbi.nlm.nih.gov/geo/query/acc.cgi?acc=GSE288068, accessed on 1 January 2025.

## Supplementary Materials

The following supporting information can be downloaded at: https://www.mdpi.com/article/10.3390/cancers17060954/s1, Figure S1: Multidimensional scaling (MDS) plots of samples used for RNA seq analysis in comparing (A) HS tumor to normal and (B) HS cell lines to control; Figure S2: A minus-average (MA) plot visualizing differential gene expression from RNA seq analysis of three HS cell lines against one control; Figure S3: Combination index (CI) plots showing the synergistic effects of combined aurora kinase inhibitor (y-axis) and trametinib (x-axis) therapy across three HS cell lines; Table S1: Sample information for 18 HS-affected and 5 control samples from canine blood and tumor tissue; Table S2: Differential gene expression data for comparison between HS tumor samples and controls; Table S3: IPA canonical pathway analysis of RNA sequencing from HS tumor samples; Table S4: IPA upstream regulator analysis of RNA sequencing from HS tumor samples; Table S5: Differential gene expression data for comparison between HS cell lines and cell strain control; Table S6: Top significantly affected signaling pathways for HS cell lines (ROSALIND); Table S7: Differential gene expression data for comparison between HS tumor samples (non-HHS) and controls; Table S8: Differential gene expression data for comparison between HHS tumor samples and controls; Table S9: ROSALIND pathway analysis of RNA sequencing from HS tumor samples using gene sets from the Pathway Interaction Database; Table S10: Differential gene expression data for comparison between HS tumor samples with a PTPN11 variant and without; Table S11: Drug assay data in HS cell lines for the aurora kinase inhibitors AT9283 and BI847325 in combination with trametinib at varying doses.
