# Supplementary material for: Transcriptome Analysis of Canine Histiocytic Sarcoma Tumors and Cell Lines Reveals Multiple Targets for Therapy
Source: Cancers (Basel). 2025 Mar 12;17(6):954. doi: 10.3390/cancers17060954 (PMC11940154; doi:10.3390/cancers17060954)
Supplement: Supplementary file 1 [file cancers-17-00954-s001.zip › Figure S1.pdf]

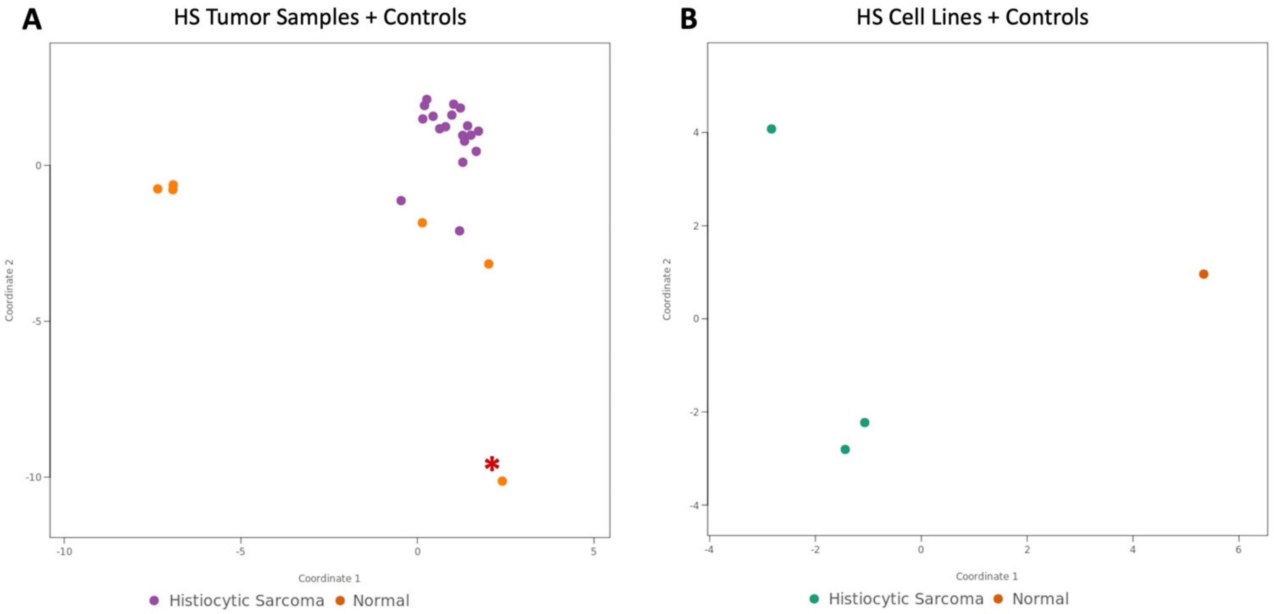

Figure S1: Multidimensional scaling (MDS) plots of samples used for RNA sequence analysis in comparing (A) HS tumor to normal and (B) HS cell lines to control. The red asterisk in panel (A), denotes an outlier sample that was excluded from further analysis.
