# Supplementary material for: Transcriptome Analysis of Canine Histiocytic Sarcoma Tumors and Cell Lines Reveals Multiple Targets for Therapy
Source: Cancers (Basel). 2025 Mar 12;17(6):954. doi: 10.3390/cancers17060954 (PMC11940154; doi:10.3390/cancers17060954)
Supplement: Supplementary file 1 [file cancers-17-00954-s001.zip › Figure S2.pdf]

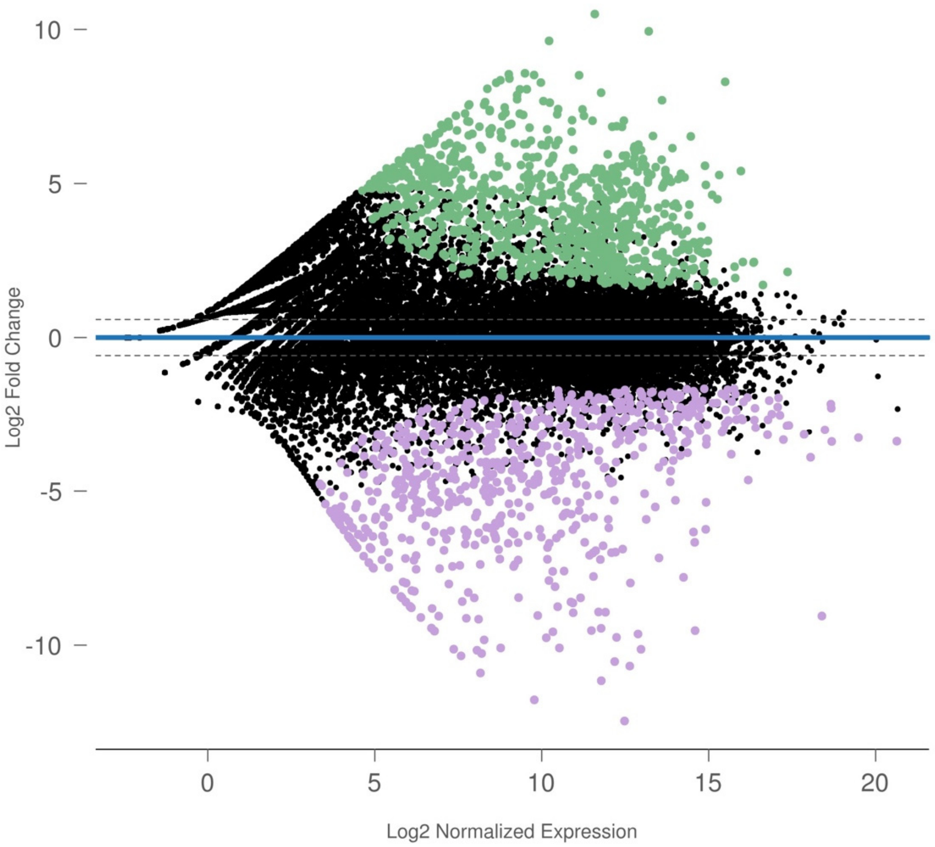

Figure S2: A minus-average (MA) plot visualizing differential gene expression from RNA sequence analysis of 3 HS cell lines (BD, OD, and PJ) against 1 control. Dots in green represent genes with significantly increased expression and dot in purple indicate decrease in gene expression. Significance was set at <0.05 p-adjusted value and a baseline of ±1.5 log2 fold change in order to capture an appropriate number of genes for pathway and gene set analysis.
