# Supplementary material for: Transcriptome Analysis of Canine Histiocytic Sarcoma Tumors and Cell Lines Reveals Multiple Targets for Therapy
Source: Cancers (Basel). 2025 Mar 12;17(6):954. doi: 10.3390/cancers17060954 (PMC11940154; doi:10.3390/cancers17060954)
Supplement: Supplementary file 1 [file cancers-17-00954-s001.zip › Figure S3.pdf]

Drug Combination	BD	OD	DH82
AT9283 + Trametinib	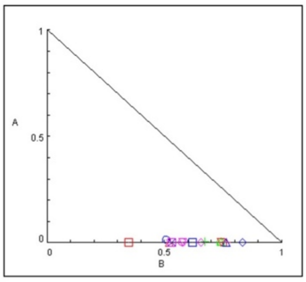	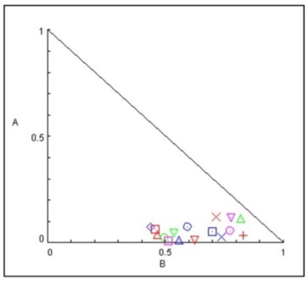	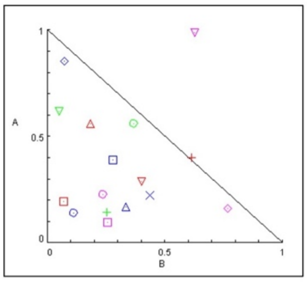
BI847325 + Trametinib	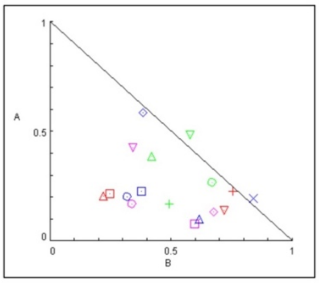	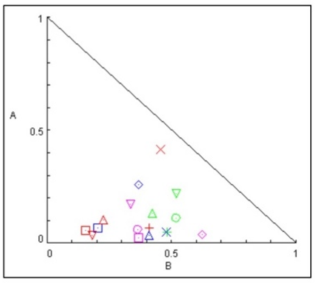	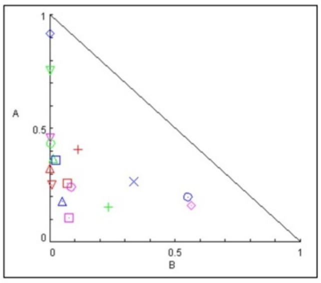

Figure S3: Combination index (CI) plots showing the synergistic effects of combined aurora kinase inhibitor (y-axis) and trametinib (x-axis) therapy across 3 HS cell lines (BD, OD, and DH82). Colored shape outlines on the plots represent different dose combinations of each set of drugs. Points below the isobole line indicate synergism, while points on the line indicate expected interaction and points above the line indicating antagonism.
