# Supplementary figures and images for: Transcriptome Analysis of Canine Histiocytic Sarcoma Tumors and Cell Lines Reveals Multiple Targets for Therapy
Source: Cancers (Basel). 2025 Mar 12;17(6):954. doi: 10.3390/cancers17060954 (PMC11940154; doi:10.3390/cancers17060954)

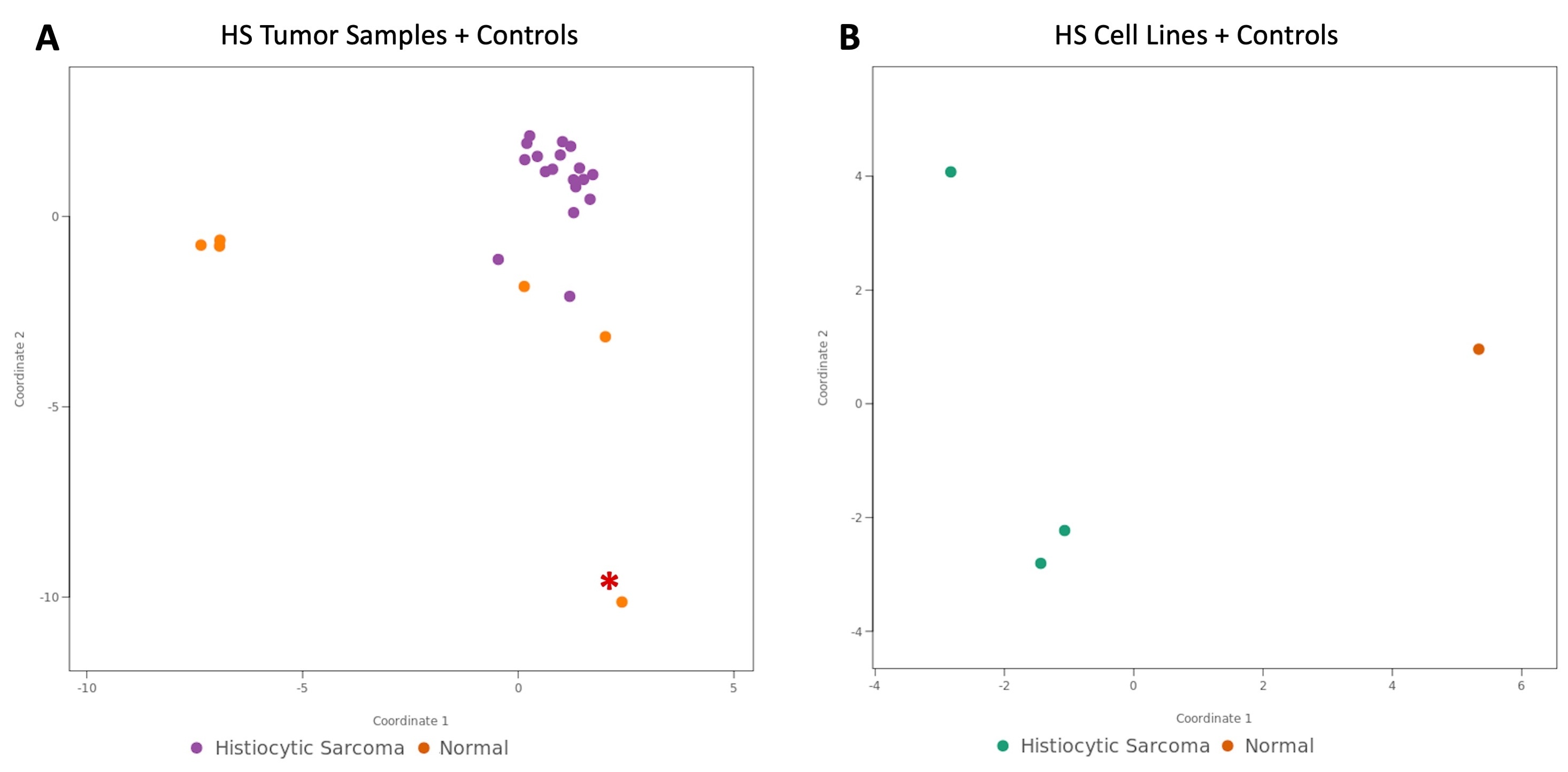

Supplement: Supplementary file 1 [file cancers-17-00954-s001.zip › Figure S1 Multidimensional scaling (MDS) plots of samples used for RNAseq analysis in comparing (A) HS tumor to normal and (B) HS cell lines to control.jpg]

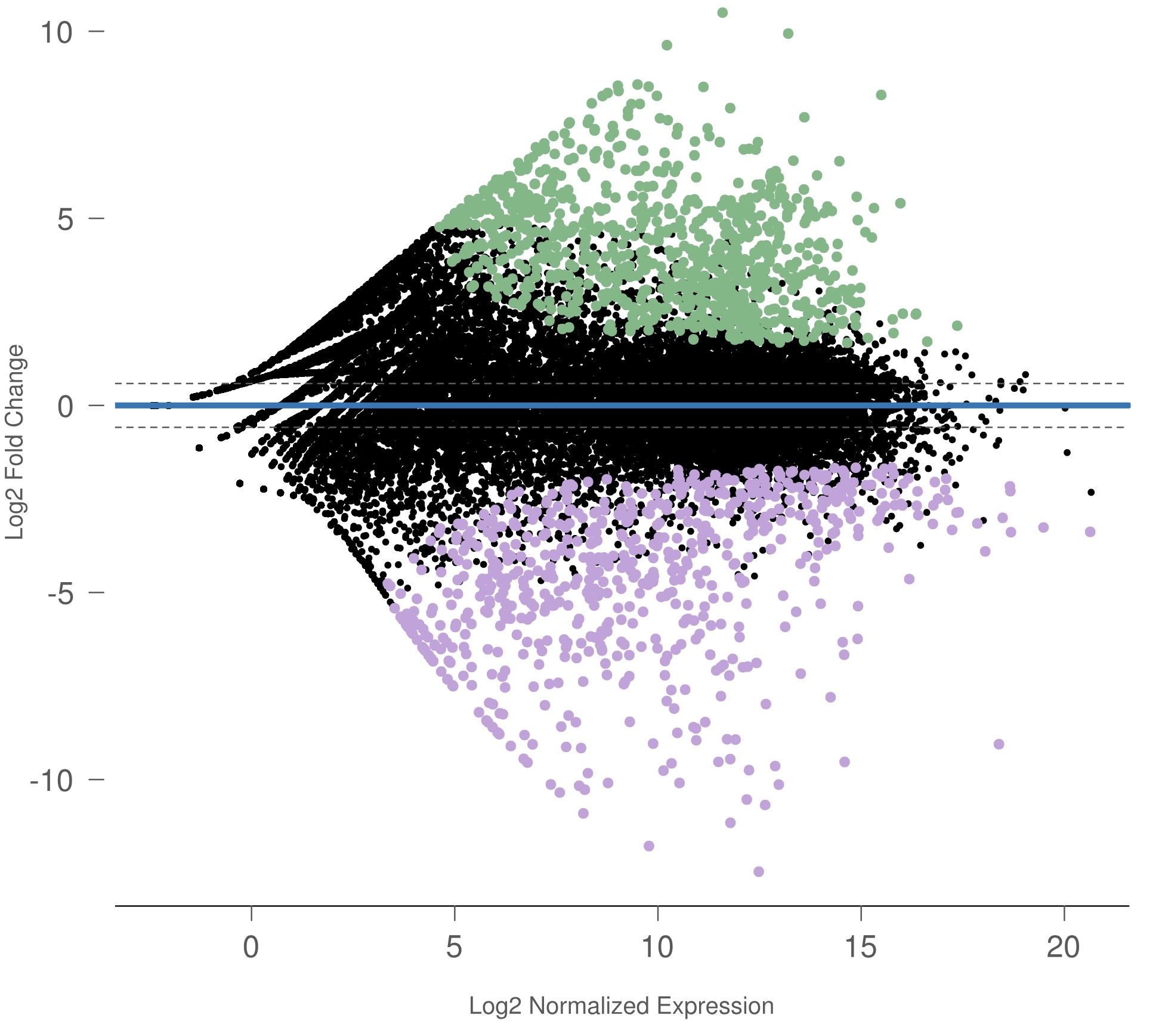

Supplement: Supplementary file 1 [file cancers-17-00954-s001.zip › Figure S2 A minus-average (MA) plot visualizing differential gene expression from RNAseq analysis of 3 HS cell lines against 1 control.jpg]

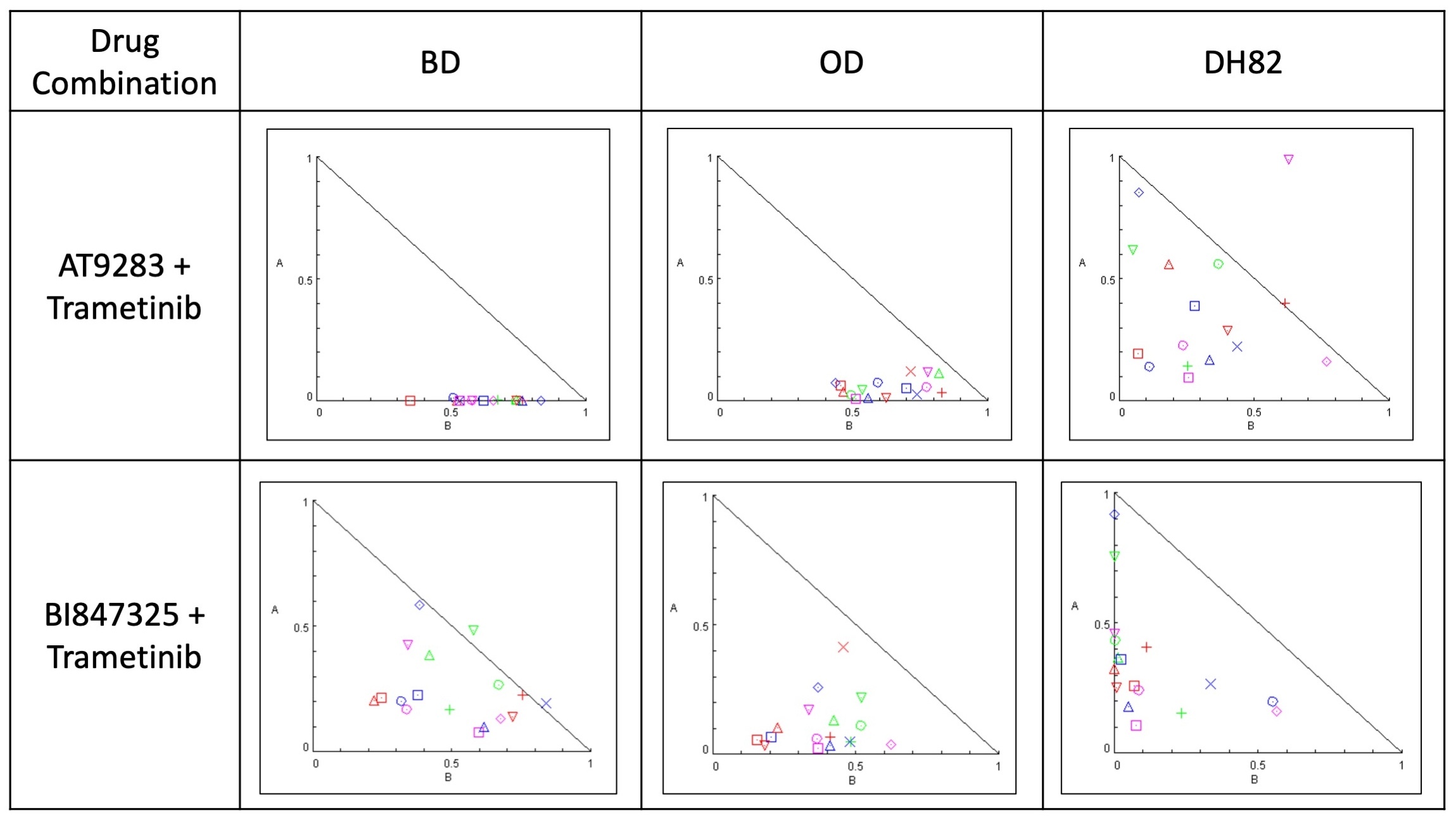

Supplement: Supplementary file 1 [file cancers-17-00954-s001.zip › Figure S3 Combination index (CI) plots showing the synergistic effects of combined aurora kinase inhibitor (y-axis) and trametinib (x-axis) therapy across 3 HS cell lines.jpg]
